# Early-season plant-to-plant spatial uniformity can affect soybean yields

**DOI:** 10.1038/s41598-022-21385-z

**Published:** 2022-10-12

**Authors:** Valentina M. Pereyra, Leonardo M. Bastos, André Froes de Borja Reis, Ricardo J. M. Melchiori, Nicolas E. Maltese, Stefania C. Appelhans, P. V. Vara Prasad, Yancy Wright, Edwin Brokesh, Ajay Sharda, Ignacio A. Ciampitti

**Affiliations:** 1 Department of Agronomy, Throckmorton Plant Science Center, Kansas State University, Manhattan, KS 66506 USA; 2 Crop and Soil Sciences Department, University of Georgia, Athens, GA 30602 USA; 3 Agricultural Center, Louisiana State University, Alexandria, LA 66506 USA; 4 INTA EEA Paraná, Ruta 11, km 12.5, 3100 Paraná, Entre Ríos Argentina; 5 Consejo Nacional de Investigaciones Científicas y Técnicas (CONICET), Buenos Aires, Argentina; 6 Facultad de Ciencias Agropecuarias, Universidad Nacional de Entre Ríos, Oro Verde, Entre Ríos Argentina; 7 John Deere, 7100 NW 62nd 18 Ave., Johnston, IA 50131 USA; 8 Department of Biological and Agricultural Engineering, Kansas State University, Manhattan, KS 66506 USA

**Keywords:** Agroecology, Ecophysiology

## Abstract

Increased soybean (*Glycine max* L. Merril) seed costs have motivated interest in reduced seeding rates to improve profitability while maintaining or increasing yield. However, little is known about the effect of early-season plant-to-plant spatial uniformity on the yield of modern soybean varieties planted at reduced seeding rates. The objectives of this study were to (i) investigate traditional and devise new metrics for characterizing early-season plant-to-plant spatial uniformity, (ii) identify the best metrics correlating plant-to-plant spatial uniformity and soybean yield, and (iii) evaluate those metrics at different seeding rate (and achieved plant density) levels and yield environments. Soybean trials planted in 2019 and 2020 compared seeding rates of 160, 215, 270, and 321 thousand seeds ha^−1^ planted with two different planters, Max Emerge and Exact Emerge, in rainfed and irrigated conditions in the United States (US). In addition, trials comparing seeding rates of 100, 230, 360, and 550 thousand seeds ha^−1^ were conducted in Argentina (Arg) in 2019 and 2020. Achieved plant density, grain yield, and early-season plant-to-plant spacing (and calculated metrics) were measured in all trials. All site-years were separated into low- (2.7 Mg ha^−1^), medium- (3 Mg ha^−1^), and high- (4.3 Mg ha^−1^) yielding environments, and the tested seeding rates were separated into low (< 200 seeds m^−2^), medium (200–300 seeds m^−2^), and high (> 300 seeds m^−2^) levels. Out of the 13 metrics of spatial uniformity, standard deviation (sd) of spacing and of achieved versus targeted evenness index (herein termed as ATEI, observed to theoretical ratio of plant spacing) showed the greatest correlation with soybean yield in US trials (R^2^ = 0.26 and 0.32, respectively). However, only the ATEI sd, with increases denoting less uniform spacing, exhibited a consistent relationship with yield in both US and Arg trials. The effect of spatial uniformity (ATEI sd) on soybean yield differed by yield environment. Increases in ATEI sd (values > 1) negatively impacted soybean yields in both low- and medium-yield environments, and in achieved plant densities below 200 thousand plants ha^−1^. High-yielding environments were unaffected by variations in spatial uniformity and plant density levels. Our study provides new insights into the effect of early-season plant-to-plant spatial uniformity on soybean yields, as influenced by yield environments and reduced plant densities.

## Introduction

Soybean (*Glycine max* L. Merril) seed costs have doubled since 1997^1^ mainly due to increased genetic yield potential^2^, the introduction of herbicide traits^3,4^, and more recently the increase in use of industrial seed treatments^5,6^. Today, farmers have adopted reduced seeding rates^7^ supported by the improved planting equipment^8^, enhanced seed quality (related to seed vigor) and seed treatments^6,9,10^, and increased yield contribution from branches in modern soybean varieties^11^. Maintaining yield while reducing seeding rates is largely associated with the growth plasticity of soybean plants^12^ (i.e., the ability of individual plants to produce compensatory yield through formation of branches). However, a plant density threshold exists, below which soybean plants cannot produce compensatory growth, and thus yield is penalized. This point is defined as the agronomical optimum seeding rate and plant density (AOSR, and AOPD, respectively)^13^. Although AOSR and AOPD are commonly assessed at the community-scale, a non-uniform plant-to-plant spacing may penalize yield at the community-scale. Therefore, early-season plant-to-plant spatial uniformity may be crucial to modern soybean production, characterized by high yield potential and reduced seeding rates^13^.

Plant-to-plant spatial uniformity seems to affect yield in soybeans mainly when the genotype by environment (GxE) interaction hinders the ability of the plants to produce compensatory yield due to limitations of resources (i.e., drought/heat stress, nutrient deficiencies, reduced growth cycle)^14–16^. For instance, soybean yield in rainfed conditions has been negatively impacted (by 6–9%) when planted in non-equidistant patterns^14–17^; however, longer maturity groups (MG) showed capacity to overcome more effectively the lack of spatial plant uniformity and yield more relative to shorter MG^14,16^. In the other hand, yield of irrigated soybeans may be maintained in both equidistant and non-equidistant plant spacings, even though plant size variability may increase^18^. These results suggest that the ability of soybean plants to produce compensatory yield is impaired by availability of resources, reducing yield in a larger proportion under non-uniform relative to uniform fields. Research into the association between spatial uniformity and soybean yield remains scarce. Improving our understanding of this relationship would substantiate the potential of precision planting techniques via their benefits on seed singulation and seeding rate recommendations, enhancing the efficiency of soybean production^19^.

The variable commonly used to assess spatial uniformity on a plant-to-plant basis is spacing between plants (average distance to neighboring plants) and is commonly summarized at the community-scale by calculating the standard deviation (sd)^18^ or coefficient of variation (cv)^14^ of distance between plants. The use of these methods to characterize plant spatial uniformity can be problematic for three reasons: (i) the spacing between plants does not account for the different distances (or uneven spacing) to each neighboring plant, or the closeness to the theoretical plant spacing (of a given seeding rate and row width); (ii) opposing plant distance outcomes (i.e., skipped and double plants) can generate similar statistical values while their effect on yield is contrasting; and (iii) standard deviation (sd)^18^ and coefficient of variation (cv)^14^ of distance between plants are strongly influenced by a few large spacings and by seeding rate (i.e. lower seeding rates result in greater spacing sd)^20^. Thus, there is a need for community-scale variables that (i) are more sensitive at detecting different spacing outcomes; (ii) provide insight into both spacing and overall distribution of plants, and (iii) are based on data collected from farmer fields that are more representative of realistic production conditions.

Following this rationale, our working hypothesis is that early-season plant-to-plant spatial uniformity is more relevant for soybean yield under limited resources (low-yielding environments) and at low seeding rates (low levels of achieved plant density). Therefore, the objectives of this study were to (i) explore both traditionally used and newly devised metrics to characterize early-season plant-to-plant spatial uniformity, (ii) identify the best metrics correlating the plant-to-plant spatial uniformity with soybean yield, and (iii) evaluate those metrics at different seeding rate (and achieved plant density) levels and yield environments.

## Materials and methods

### Sites description and field operations

A total of six field studies were conducted in two different regions over two seasons. Four studies (two dryland and two irrigated) were in Kansas, United States (dryland: 39°4′30″ N, − 96°44′43″ W, irrigated: 39°4′25″N, − 96°43′12″ W) during the 2019 and 2020 growing seasons (hereafter referred to as USDry19, USIrr19, USDry20, and USIrr20 studies). The remaining two studies (dryland) were in Entre Rios, Argentina (31°50′49″ S; 60°32′16″ W) during the 2018/2019 and 2019/2020 growing seasons (hereafter referred to as Arg19 and Arg20 studies). The soils were Fluventic Hapludolls [silt loam, 40% sand, 13% clay, 47% silt, organic matter (OM) 1.7%, 7.7 pH, 31.1 ppm P (Bray^−1^)] at the US dryland studies, and Pachic Argiudolls [silty clay loam, 10.1% sand, 30.6% clay and 59.3% silt, OM 3.2%, 6.8 pH, 34.7 ppm P (Bray^−1^)] at the US irrigated studies. At the Argentinian studies soil was a Vertic Argiudoll in 2019 [silty clay loam to clay loam, 3.9% sand, 27.6% clay, 67.9% silt, OM 2.65%, 7.2 pH, 12.5 ppm P (Bray^−1^)] and an Acuic Argiudoll in 2020 [silt loam to silty-clay-loam, 5.6% sand, 28.6% clay, 65.8% silt, OM 3.33%].

The US dryland and irrigated studies were sown on June 4, 2019, and May 20, 2020. In 2019, the dryland study was replanted on June 29 due to poor emergence after the first sowing. The studies in Argentina were sown on December 5 in 2018 and November 20 in 2019. At all six studies, plots were kept free of weeds, pests, and diseases through recommended chemical control.

The genotypes used in the US were P40A47X (MG 4.0) and P39A58X (MG 3.9) (Corteva Agriscience, Johnston, IA, USA) in 2019 and 2020, respectively. Both varieties are tolerant to glyphosate and dicamba herbicides (RR2X) and have low lodging probability. For the northeast region of Kansas, recommended sowing dates range from May 15 to June 15 along with MG 4^21^. In addition, recommended seeding rates are between 270 and 355 thousand seeds ha^−1^ for low-yielding environments and 190 to 285 thousand seeds ha^−1^ for medium- and high-yielding environments^13^. In Argentina, the genotype AW5815IPRO (MG 5.8, Bayer, Leverkusen, Germany) was used both in 2020 and 2021, it is tolerant to glyphosate and sulfonylureas, and has low lodging probability. Recommended sowing dates for Entre Rios considering soybeans as a single crop range from October 20 to December 10, and MG usually range from 4 to 6; lastly, seeding rate recommendations are between 200 and 250 thousand seeds ha^−1^ in the region^22^.

### Study design

The studies carried out in the US were arranged as a split plot design with three replicates in both 2019 and 2020. In 2019, the main plot treatment factor was planter type with two levels [John Deere (Moline, Illinois, US) Max Emerge planter (ME, 12 rows), and John Deere Exact Emerge Planter (EE, 16 rows)], and the split-plot treatment factor was seeding rate with two levels (160 and 321 thousand seeds ha^−1^). In 2020 the main plot treatment factor was also planter type with two levels (ME and EE), and the split-plot treatment factor was seeding rate with four levels (160, 215, 270 and 321 thousand seeds ha^−1^). Planting speed was 7 km h^−1^ in both studies and years, plots were 24 and 32 rows wide when planted with ME and EE, respectively, with 0.76 m row spacing. Plot length was 80 m in the dryland studies and 160 m in the irrigated studies. The studies in Argentina were arranged as a single replicate of each seeding rate (100, 230, 360 and 550 thousand seeds ha^−1^) in both years. Planting speed was 5.5 km h^−1^ in both years, and plots were 10 rows wide with 0.52 m row spacing and 350 m in length.

All treatment factors in US studies were evaluated with the overall goal of producing substantial variation in the variable of interest, plant-to-plant spatial uniformity, rather than to make an inference of their effect on yield. The Argentinian studies were only used for selection of stand uniformity variables due to the single replicate. Plant spatial uniformity variables were first fitted using the data from US studies (details below), and then the best explanatory metrics were selected to re-fit the relationships combining both data sets from US and Argentina. Finally, sowing dates, maturity groups, and seeding rates evaluated in this study at both locations (Arg and US) were aligned with those recommended for each region.

### Data collection and spacing uniformity variables

Two segments of 2 m in length were established early in the season inside each plot. At the V5 (US studies) and R1 (Arg studies) soybean development stage^23^, the cumulative distance of the plants within each segment was measured and then used to calculate multiple derived variables. Plant spacing (cm) was calculated as the average distance between neighboring plants. In addition, the distance from a plant to each neighboring plant was classified as shorter or longer than the plant spacing (named nearest and farthest neighbor distance, respectively). Achieved versus Target Evenness Index (ATEI, dimensionless) was calculated as the ratio between the observed plant spacing and the theoretical plant spacing (TPS, cm), where TPS is the expected plant spacing derived from a specific seeding rate and row width (Eq. 1).

1 $$ATEI = \frac{Spacing\;(cm) }{{TPS\;(cm)}}$$

The ATEI index was designed to account for the proximity of the observed plant spacing to the TPS. Values closer to 1 indicate that the plant spacing is close to the TPS and values that are below or above 1 indicate that the plant spacing is lower or higher than the TPS, respectively; thereby departing from an ideal plant spacing. Hence, ATEI values greater than 1 depict both (i) non-uniform plant-to-plant spacing distribution and (ii) plant densities below the target (seeding rate). To further understand the meaning of ATEI, the relative density (rd) was calculated as the ratio between plant density (based on the number of plants in the 2 m segment) and seeding rate.

To account for the unevenness of distance from a plant to both neighboring plants within the row, we used the Evenness Index (EI, dimensionless), calculated as the ratio between the distance to the nearest neighbor (cm) and the plant spacing (cm) of a given plant (Eq. 2). The Evenness Index values range from 0 to 1, a value closer to 1 indicates that a plant is equidistantly spaced to both of its neighboring plants within the row, if zero then those plants are occupying the same position (as doubles). It is important to note that EI does not provide information on the spacing (in distance, cm) or how close the spacing is compared to the TPS, but only describes the unevenness distance of a plant to its neighboring plants within a row.

2 $$Evenness \;Index\; (EI) = \frac{nearest\; neighbor \;(cm)}{{Spacing\; (cm)}}$$

**Figure 1:**
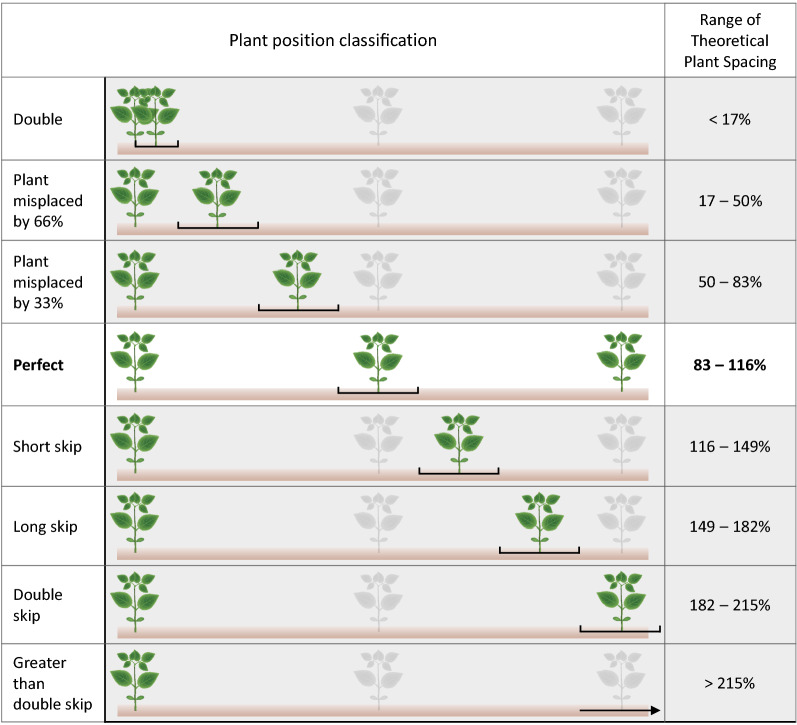
Plant position classification based on the observed spacing to the previous plant within a row compared to the theoretical plant spacing (TPS). The TPS is the targeted (perfect) plant spacing of a plant population given a seeding rate and row width. The difference between TPS and observed spacing to the previous plant was rated as double (< 17.5% of TPS); misplaced by 66% (between 17.5–50.5% of TPS), misplaced by 33% (between 50.5–83.5% of TPS), perfectly spaced plants (between 83.5–116.5% of TPS), short-skip plant (between 116.5–149.5% of TPS), long-skip plants (between 149.5–182.5% of TPS), double-skip plants (between 182.5–215.5% of TPS), and greater than double-skip plants (> 215.5% of TPS).

In addition, the distance from a plant to its preceding neighboring plant, and the TPS were used to classify the position of each plant into one of eight classes (Fig. 1). Plants were classified in classes ranging from “double” (preceding plant distance < 17.5% of TPS), to “perfect” (preceding plant distance between 83.5 and 116.5% of TPS), and “greater than double skip” (preceding plant distance greater than 215.5% of TPS).

Plant spatial uniformity metrics (Eqs. (1) and (2), Fig. 1) were calculated on a plant-to-plant basis and then summarized at the segment level (hereafter called community-scale). Plant spacing (cm), ATEI, and EI were summarized by calculating their mean (except for plant spacing) and sd. Plant spacing sd and cv (directly proportional to sd) have been used in previous studies^14,18^ to estimate spatial variability. Hence, plant spacing sd (cm) was included as the base line variable to compare the performance of the new metrics proposed in this study. Lastly, plant position classes (Fig. 1) were summarized by calculating the mean percentage of plants in a segment classified within each of the eight categories.

At physiological maturity, 10 (US studies) or 20 (Arg studies) consecutive plants within each segment were hand cut, individually identified, bagged, and oven-dried at 65 °C until constant weight was achieved. Grain weight per plant was recorded and is reported on a 13 g kg^−1^ water content. Yield (Mg ha^−1^) was calculated at the segment level by summing the grain weight (g) of all harvested plants and correcting it to the segment area.

### Statistical analysis and software

Community-scale data from the four US studies (site-years) were combined to model yield and the 13 stand uniformity metrics (ATEI mean, ATEI sd, EI mean, EI sd, Spacing sd (cm), Double, Mis 33, Mis 66, Perfect, Short-skip, Long-skip, Double-skip, > Double-skip) as a function of seeding rate, planter type and their interaction (fixed effects), and block nested in site-year (random effect) (Tables 1 and 2). Independent models for each of the 4 US studies were built assessing the effects of planter type, seeding rate, and their interaction (fixed effects), and seeding rate nested in planter type, and in block (random effects) on the same variables previously mentioned (Supplementary Table 1). The models were run using the lmer function from *lme4* package in R (R Core Team, 2021). In addition, the US and Arg studies were combined to evaluate the effect of site-year on yield, plant density, and all stand uniformity variables (Supplementary Fig. 1) using the lm function from package *stats*. Means separation were performed using Fisher’s LSD (Least Significance Difference) test (alpha = 0.05) with emmeans function from package *emmeans*.

**Table 1:** Effect of planter type, seeding rate, and their interaction on variables from plant position classification for all US studies. References: percentage of perfectly spaced plants (Perfect), percentage of plants misplaced by 66% (Mis 66), percentage of plants misplaced by 33% (Mis 33), percentage of double plants (Double), percentage of short skips plants (Short-skip), percentage of long skip plants (Long-skip), percentage of double skips plants (Double-skip), and percentage of greater than double skip plants (> Double-skip).

	Perfect (%)	Mis 66 (%)	Mis 33 (%)	Double (%)	Short skip (%)	Long skip (%)	Double skip (%)	> Double skip (%)
**Planter**^**1**^								
EE	23.8 a^2^	4.3 b	15.5	1.3 b	17.0	11.4	8.1	18.9
ME	11.8 b	13.1 a	12.2	7.8 a	14.1	9.1	8.5	23.7
p-value	*< 0.001*	*< 0.001*	*0.070*	*< 0.001*	*0.126*	*0.165*	*0.812*	*0.057*
**Seeding rate (thousand seeds ha**^**−1**^**)**								
161	22.7 a	6.4	12.7	3.9 ab	15.7	9.0 ab	6.8	22.3
215	21.1 a	9.6	10.6	3.9 b	17.0	13.1 a	7.9	17.7
269	13.2 b	10.0	16.5	3.1 b	13.7	13.1 a	10.0	21.5
321	14.1 b	8.9	15.6	7.0 a	16.0	5.8 b	8.5	23.8
p-value	*< 0.001*	*0.168*	*0.108*	*0.023*	*0.766*	*0.003*	*0.445*	*0.493*
Planter x Seeding rate	0.011	0.085	0.414	0.249	0.032	0.122	0.787	0.885

^1^Planter type: Max Emerge (ME) and Exact Emerge (EE).

^2^Letter and ANOVA (analyses of variance) threshold. Different letters mean significant differences among treatments.

The values in italics correspond to the p-values.

**Table 2:** Effect of planter type, seeding rate, and their interaction on yield and stand uniformity variables for all US studies. References: Spacing between plants standard deviation (Spacing sd), achieved versus targeted evenness index mean and standard deviation (ATEI and ATEI sd, respectively), and evenness index mean and standard deviation (EI and EI sd, respectively).

	Yield (Mg ha^−1^)	Spacing sd (cm)	ATEI	ATEI sd	EI	EI sd
**Planter**						
EE	3.19	4.0	1.6	0.7	0.7 a	0.2 b
ME	3.2	4.5	1.5	0.8	0.5 b	0.3 a
p-value	*0.948*	*0.371*	*0.489*	*0.383*	*< 0.001*	*< 0.001*
**Seeding rate (thousand seeds ha**^**−1**^**)**						
161	3.34 a	5.2	1.5	0.6 b	0.6 a	0.2 b
215	3.36 ab	4.1	1.5	0.7 b	0.6 a	0.2 b
269	3.06 ab	3.3	1.5	0.7 b	0.6 ab	0.2 b
321	3.02 b	4.4	1.7	1.1 a	0.5b	0.3 a
p-value	*0.04*	*0.058*	*0.051*	*< 0.001*	*< 0.001*	*0.01*
Planter x Seeding rate	0.831	0.978	0.760	0.977	0.229	0.020

^1^Planter type: Max Emerge (ME) and Exact Emerge (EE).

^2^Letter and ANOVA threshold. Different letters mean significant differences among treatments.

The values in italics correspond to the p-values.

**Figure 2:**
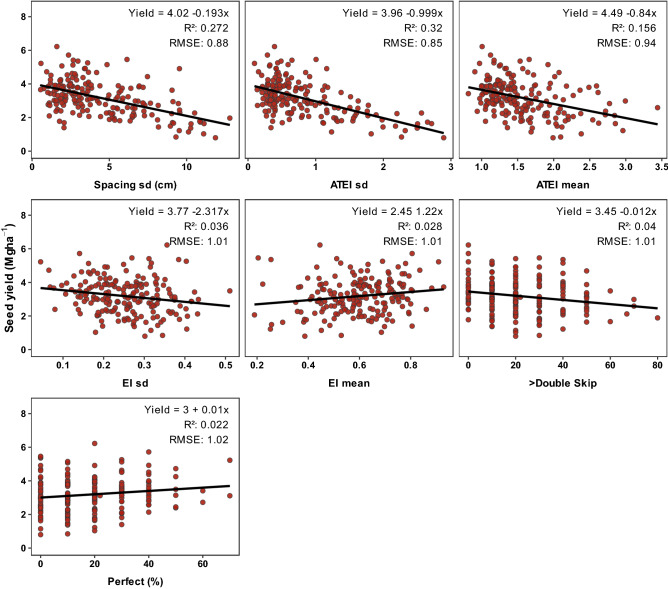
Relationship between stand uniformity variables and soybean yield for US studies. *ATEI mean and sd* achieved versus targeted evenness index mean and standard deviation, *EI mean and sd* evenness index mean and standard deviation, *Perfect* percentage of perfectly spaced plants, *R*^*2*^ coefficient of determination, *RMSE* root mean square error. All stand uniformity variables presented a significant slope at alpha = 0.05.

**Figure 3:**
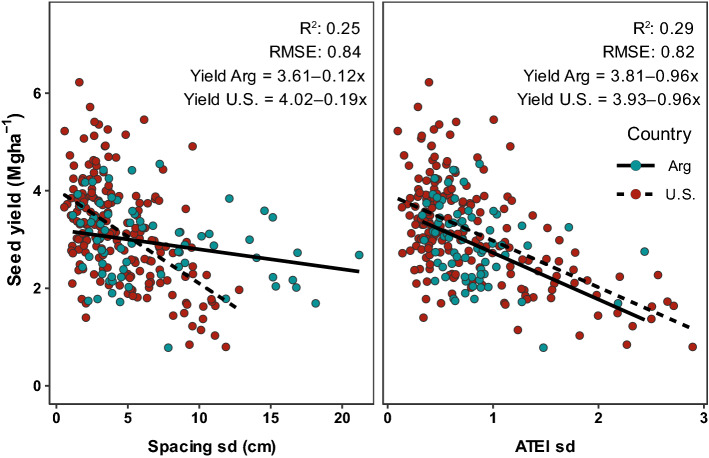
Relationship of spacing standard deviation (Spacing sd, cm) and achieved versus targeted evenness index standard deviation (ATEI sd) to soybean yield. Different colors and line types denote different countries (Argentina, Arg—full line, red points; United States, US—dashed line, blue points). *R*^*2*^ coefficient of determination, *RMSE* root mean square error.

Community-scale data from the four US studies were combined and fitted to bivariate linear regression models with yield as the response variable and each of the stand spatial uniformity variables as the explanatory variable. Significant models (alpha = 0.05) were further evaluated by calculating the coefficient of determination (R^2^) and root mean squared error (RMSE) (Fig. 2). Models with the lower RMSE and higher R^2^ were selected as those that best captured the effect of non-uniform stands on soybean yield. After variables were selected, both US and Arg data sets were combined and the linear regressions between the selected variables and yield were re-fitted to assess the consistency of the relationships when an independent data set was included. Community-scale yield from US and Arg studies was modelled as a function of the selected stand uniformity variable, country (US and Arg), and their interaction (fixed effects) (Fig. 3). The spatial uniformity metric showing the most consistent relationship for both US and Arg studies (i.e., non-significant interaction between stand uniformity metric and country), was selected to continue the analysis. The bivariate linear regression models were run with function lm.

Different environmental conditions and seeding rate levels may modify the effect of plant spatial uniformity on yield. To explore this, each of the studies from Arg and US were separated into low- (USDry19 and ArgDry20, mean of 2.7 Mg ha^−1^), medium- (USIrr19, USDry20 and ArgDry19, mean of 3.0 Mg ha^−1^), and high- (USIrr20, mean of 4.3 Mg ha^−1^) yield environments based on the effect of site-year on yield (Supplementary Fig. 1). Additionally, the tested seeding rates were separated in low (< 200 thousand seeds ha^−1^), medium (between 200 and 300 thousand seeds ha^−1^) and high (> 300 thousand seeds ha^−1^) levels based on the current optimal seeding rate for medium yielding environments (235 thousand seeds ha^−1^, 4 Mg ha^−1^)^13^ and the extreme values proposed by Suhre et al.^11^ (148 and 445 thousand seeds ha^−1^). This classification was used to model yield as a function of (i) the selected stand uniformity metric, yield environment, and their interaction, and (ii) the selected stand uniformity metric, seeding rate levels, and their interaction. These models were tested to obtain a robust conclusion on the overall effect of yield environment and seeding rate levels, and their interactions (all treated as fixed effects) with plant-to-plant spatial uniformity relative to the response variable, soybean yield. The Akaike information criteria (AIC) was used to compare the full (with interactions) relative to the reduced models (single effects).

### Ethics declarations

Experimental research and field studies on plants including the collection of plant material, complied with relevant institutional, national, and international guidelines and legislation.

## Results

### Treatment effects on yield and spatial stand uniformity metrics

Mean cumulative seasonal precipitation was 560 and 630 mm for the USDry19 and USDry20, respectively; and 770 and 450 mm for the Arg19 and Arg20, respectively. Total water availability was 712 mm for USIrr19 (152 mm from irrigation) and 757 mm for USIrr20 (127 mm from irrigation). The effect of the treatments analyzed in this study (planter type and seeding rate) were significant on yield and in 8 out of 13 stand uniformity metrics (alpha = 0.05, Tables 1 and 2). From the four seeding rates evaluated, attention will be given only to the lowest (161 thousand seeds ha^−1^) and highest (321 thousand seeds ha^−1^) seeding rates as only those levels were present at all four US studies. Yield was not affected by planter type, but a significant effect of seeding rate was observed. Yield was greater at the low seeding rate (3.3 Mg ha^−1^) compared to the high seeding rate (3.0 Mg ha^−1^). In terms of the spatial uniformity metrics, the EE planter generally improved the uniformity between plants compared to ME planter. The EE planter increased the number of perfectly spaced plants (24%), decreased the number of double plants (1.3%), and plants misplaced by 66% (4.3%) compared to the ME planter (12, 7.8, and 13% respectively). In addition, EE achieved a higher mean evenness index (EI) and lower evenness index sd (0.7 and 0.2, respectively) compared to ME (0.5 and 0.3, respectively). In terms of the variables affected by seeding rate, the low seeding rate was associated with a greater proportion of perfectly spaced plants (23%), fewer double plants (4%), smaller ATEI sd and evenness index sd (0.6 and 0.2, respectively), and an increased mean evenness index (0.6) compared to that associated with the high seeding rate (14 and 7.0% of perfectly spaced and double plants, respectively; 1.1, and 0.3 for ATEI sd and evenness index sd, respectively; and 0.5 for mean evenness index). Thus, the lower seeding rate resulted in more uniform spacing between plants.

Average yield was greatest in the USIrr20 (4.3 Mg ha^−1^, high yield environment), medium in Arg19, USIrr19 and USDry20 (2.9 to 3.2 Mg ha^−1^, medium yield environment), and lowest in the Arg20 and USDry19 (2.8 and 2.6 Mg ha^−1^, respectively, low yield environment). More information on individual studies and treatment effects on yields can be consulted in the supplementary material (Supplementary Table 1, Supplementary Fig. 1).

### Spatial stand uniformity effects on soybean yield

Soybean yield from the four US studies (n = 190) was significantly correlated with 7 out of the 13 plant spatial uniformity metrics (alpha = 0.05, Fig. 2). Yield was negatively correlated with ATEI sd (R^2^ = 0.32), Spacing sd (cm, R^2^ = 0.27), ATEI mean (R^2^ = 0.16), > Double-skip plants (%, R^2^ = 0.04), and Evenness Index (EI, R^2^ = 0.04) sd. While soybean yield was positively correlated with EI mean (R^2^ = 0.03) and Perfectly spaced plants (R^2^ = 0.02). Spacing sd and ATEI sd were selected to continue with the analysis due to their greater R^2^ and lower RMSE.

Spacing sd and ATEI sd, the selected metrics based on R^2^ and RMSE (Fig. 2), were correlated with yield from all six studies (US and Arg, n = 262). Yield from Arg was significantly correlated with both spacing sd and ATEI sd. However, only ATEI sd presented a stable relationship across datasets of both countries (non-significant interaction). In this sense, for each unit of increase in spacing sd, US showed a greater reduction in yield (0.11 Mg ha^−1^) compared to Arg (0.03 Mg ha^−1^), even though the observed range of spacing sd was smaller in US (0.5–12.8 cm) than Arg (1.2–21.7 cm). This indicates greater variability in the spacing between plants in Arg did not translate to a greater yield reduction relative to the US dataset. Whereas ATEI sd and yield relationship had a different intercept (due to yield levels US 3.8, and Arg 3.7 Mg ha^−1^), the slope of yield decreased per unit increase in ATEI sd did not statistically differ between datasets (0.96 Mg ha^−1^, Fig. 3). Moreover, ATEI sd ranges were similar between the US (0.1–2.9) and Arg (0.3–2.4). Thus, ATEI sd was selected as the variable that best explained plant spatial uniformity effects on soybean yield.

**Figure 4:**
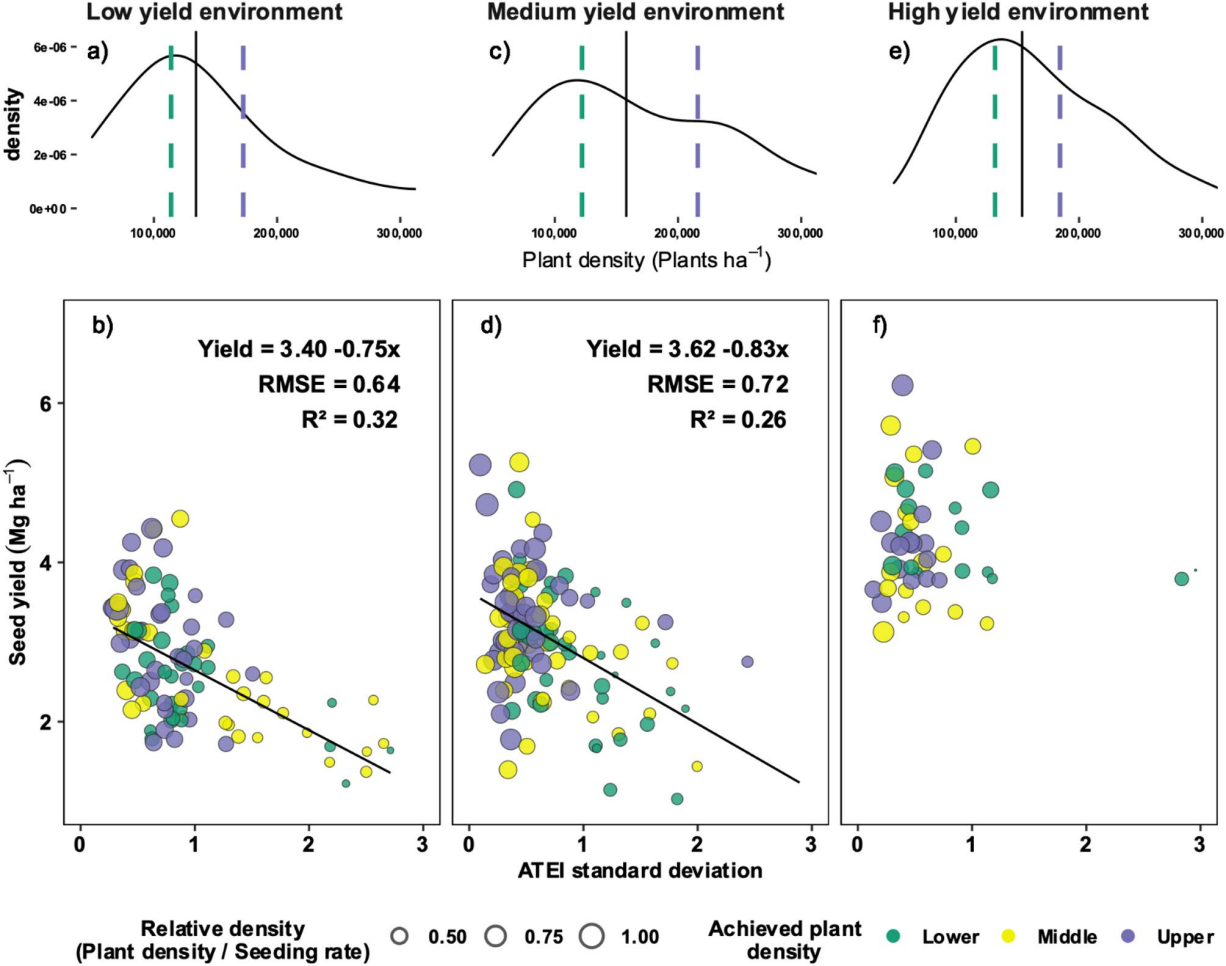
Relationship between soybean yield (Mg ha^−1^) and ATEI sd (achieved versus targeted evenness index standard deviation) in low- (**b**, mean of 2.7 Mg ha^−1^), medium- (**d**, mean of 3.0 Mg ha^−1^), and high- (**f**, mean of 4.3 Mg ha^−1^) yield environments. The size of the bubbles indicates the relative density (plant density to seeding rate ratio), and colors indicate lower (red), middle (blue), and upper (green) thirds of achieved plant density distribution within each yield environment. Panels (**a**), (**c**), and (**e**) show the density distribution of the obtained plant density (plants ha^−1^) by yield environment, full line and dashed lines denotes the median, and lower–upper terciles, respectively. Yield environments are based on ANOVA of site-year versus yield (Supplementary Fig. 1). Interaction between ATEI sd and yield environment was significant (alpha = 0.05). *R*^*2*^ coefficient of determination, *RMSE* root mean square error.

To assess the relationship between yield and plant spatial uniformity (ATEI sd) at different yield environments, site years were divided into Low (USDry19 and Arg20, 2.7 Mg ha^−1^), Medium (USIrr19, Arg19, and USDry20, 3.0 Mg ha^−1^), and High (USIrr20, 4.3 Mg ha^−1^) yield environments (Supplementary Fig. 1). Increases in ATEI sd negatively impacted yield in both low- and medium- yield environments (0.75 and 0.83 Mg ha^−1^ per unit increase in ATEI sd, respectively), while no effect was observed in high-yield environments (p > 0.05, Fig. 4). Moreover, yield from low- and medium- yield environments showed a strong correlation with ATEI sd (R^2^ of 0.32 and 0.26, respectively). Although the relationship between yield and plant spatial uniformity (ATEI sd) did not change with seeding rate levels (Supplementary Table 2), the achieved plant density changed across studies. The achieved plant densities (panels a, c, and e in Fig. 4) were similar across yield environments with a median of 148 thousand plants ha^−1^. Yield reductions in low- and medium-yield environments were associated with values of ATEI sd > 1, plant densities < 200 thousand plants ha^−1^, and with achieved relative to target densities (bubble sizes, panels b, d, and f in Fig. 4) around 50%. The model performance (measured by AIC) improved by including the effect of yield environment relative to the simpler model (ATEI sd only).

## Discussion

This study provides new insights into the relevance of soybean plant-to-plant spacing uniformity, mainly under low- and medium-yield environments and reduced plant densities. Soybean breeding efforts have altered the overall contribution of branches to yield, reducing the dependency on high seeding rates over time^11,12^. Still, low yielding environments limit the ability of the plants to produce compensatory yield from branches and thus require increased seeding rates^13,24^. Our results showed that in both high- and low-yield environments, soybean yield was not increased by seeding rates greater than 121 thousand seeds ha^−1^. Instead, yield variability from low- and medium-yield environments was explained by plant spatial uniformity (ATEI sd).

Plant-to-plant spatial uniformity effects on yield can be understood from the onset of competition at the plant level, which determines yield at the community-scale. Soybean seedlings initiate shade avoidance responses when they sense reductions in the red:far red ratio of the light spectrum (i.e., shading due to increased plant-to-plant competition), causing reductions in branch development, overall growth, and yield per plant^25–27^. Similarly, a reduction of early-season plant-to-plant spatial uniformity could result in a continuum of shade avoidance responses with differing competitive abilities and yields at the plant level. At a community-level, the ability of soybean plants to produce compensatory yields via branches^28,29^ is impaired by restrictive environments such as late planting dates^30^ or drought stress^29^. In summary, early-season plant-to-plant uniformity can determine the onset of a differential competitive ability among individual plants^31^ mainly under limited resources and reduced plant densities penalizing yield at a community scale.

The most promising metric of plant spatial uniformity, ATEI sd, explained the highest variability of soybean yield (higher R^2^) and reduced the error of the estimation (smaller RMSE) across studies from the US and Arg (Fig. 3). Furthermore, the indices of stand uniformity presented (Fig. 1, Eqs. (1), (2)) were able to detect differences in spatial distribution between planter types and among seeding rates, whereas the most common uniformity index used in previous literature, spacing sd, did not (Tables 1 and 2). A previous study has shown that 77% of spacing sd variability is explained by clusters (3 or more plants together), double plants, short-skips, and long-skips, with the latter making the greatest contribution to its variability^32^. High spatial stand uniformity of seedlings is dependent not only on a highly uniform sowing pattern, but also on high emergence ratios^33^. In this sense, ATEI sd reflects non-uniform spacing as well as poor emergence and plant mortality. In summary, ATEI sd is a better predictor of soybean yield and is a useful metric for measuring within-row stand uniformity in future research.

This study is a first attempt at describing the effects of spatial uniformity on soybean yield in environments producing phenotypes that do not express the plant’s growth potential, penalizing yield at the community-scale. In agreement, a review from Lu et al.^33^ concluded that plant spatial uniformity resulting from precision sowing is an effective way to increase soybean yields at all seeding rates. Moreover, lowering seeding rates along with improvements in planting quality delivering seed at a uniform spacing and depth can become critical components of modern soybean systems. Farmers could adopt such practices as an insurance against yield losses in poor-yielding conditions. Future studies exploring AOSR in low- and medium- yielding environments should consider plant spatial uniformity and relative density (plant density to seeding rate ratio)^13,24^ as factors that may confound the yield response to changes in seeding rates. Improved foundational knowledge of key soil and environmental factors affecting early- and within-season plant-to-plant spatial uniformity is still a research gap that should be explored in future research studies. Finally, the study of the differential plant-to-plant ability to produce compensatory yield^31^ could clarify the influence of canopy characteristics determined early in the season on yield formation under realistic field conditions.

## Conclusion

Our working hypothesis “early-season spatial plant-to-plant uniformity is more relevant for soybean yield under limited resources (low yielding environments) and under low seeding rates (low levels of achieved plant density)” is partially accepted. On one side, early-season plant-to-plant spatial uniformity impacted soybean yields mainly under both low- and medium-yield environments. However, this effect is not only relevant for low seeding rates, but mainly for plant densities below 200 thousand plants ha^−1^. Plant spatial uniformity under field conditions results from uniformity in the sowing pattern, emergence, and early-establishment of plants. To capture these effects, relating the observed spacing with the TPS (obtaining ATEI) removed the confounding effects of spacing sd. The negative impact on soybean yields due to reduced spatial uniformity and plant establishment suggests an impairment of plants to produce compensatory yields under resource-limited environments. Hence, yield optima in low- and medium-yield environments resulted from improved plant spatial uniformity (ATEI sd < 1) and plant densities above 200 thousand plants ha^−1^.

## Supplementary Information

Supplementary Information.

## Data Availability

The data that supports the findings of this study are available from the authors, upon reasonable request.
