# Supplementary material for: Early-season plant-to-plant spatial uniformity can affect soybean yields
Source: Sci Rep. 2022 Oct 12;12:17128. doi: 10.1038/s41598-022-21385-z (PMC9556638; doi:10.1038/s41598-022-21385-z)
Supplement: Supplementary file 1 — Supplementary Information. [file 41598_2022_21385_MOESM1_ESM.docx]

**Supplementary Material**

**Supplementary Table 1.** Effect of planter type, seeding rate, and their interaction for each of the four US site years. References: Spacing between plants standard deviation (Spacing sd), percentage of perfectly spaced plants (Perfect), percentage of plants misplaced by 66% (Mis 66), percentage of plants misplaced by 33% (Mis 33), percentage of double plants (Double), percentage of short skips plants (Short-skip), percentage of long skip plants (Long-skip), percentage of double skips plants (Double-skip), percentage of greater than double skip plants (> Double-skip), achieved versus targeted evenness index mean and standard deviation (ATEI and ATEI sd, respectively), and evenness index standard deviation (EI sd).

| **USIrr19** |  | **Yield (Mg ha^-1^)** | **Spacing sd (cm)** | **Perfect (%)** | **Mis 66 (%)** | **Mis 33 (%)** | **Double (%)** | **Short skip (%)** | **Long skip (%)** | **Double skip (%)** | **> Double skip (%)** | **ATEI** | **ATEI sd** | **EI** | **EI sd** |
| --- | --- | --- | --- | --- | --- | --- | --- | --- | --- | --- | --- | --- | --- | --- | --- |
| **Planter^1^** | **ME** | 3.01 | 5.8 | 14.2 | 2.9 a^2^ | 11.3 | 2.5 b | 10.4 a | 10.4 | 10.4 | 37.9 a | 2.1 a | 1.0 | 0.6 a | 0.2 b |
| **(P)** | **EE** | 2.88 | 5.8 | 10.0 | 11.2 a | 10.8 | 10.8 a | 17.9 a | 7.9 | 6.7 | 24.6 a | 1.5 b | 1.0 | 0.5 a | 0.3 a |
|  | *p-value* | *0.69* | *0.975* | *0.465* | *0.002* | *0.879* | *<0.001* | *0.009* | *0.411* | *0.192* | *0.004* | *<0.001* | *0.825* | *0.001* | *<0.001* |
| **Seeding rate** | **161** | 3.04 | 5.0 | 6.67 b | 10.0 a | 11.7 | 7.5 | 13.8 | 6.3 | 9.6 | 34.6 | 1.9 | 1.23 a | 0.5 a | 0.3 a |
| **(k seeds ha^-1^) (SR)** | **321** | 2.84 | 6.6 | 17.5 a | 4.2 a | 10.4 | 5.8 | 14.6 | 12.1 | 7.5 | 27.9 | 1.7 | 0.80 a | 0.6 a | 0.3 a |
|  | *p-value* | *0.548* | *0.124* | *<0.001* | *0.029* | *0.649* | *0.362* | *0.772* | *0.055* | *0.468* | *0.149* | *0.05* | *0.023* | *0.007* | *0.016* |
| **P x SR** |  | *0.726* | *0.567* | *0.357* | *1.000* | *0.448* | *0.362* | *0.246* | *1.000* | *0.664* | *0.857* | *0.342* | *0.566* | *0.164* | *0.012* |
|  |  |  |  |  |  |  |  |  |  |  |  |  |  |  |  |
| **USDry19** |  |  |  |  |  |  |  |  |  |  |  |  |  |  |  |
| **P** | **ME** | 2.66 | 6.6 | 10.4 a | 7.50 a | 13.3 | 12.2 a | 9.6 a | 7.5 | 6.3 | 33.2 a | 1.9 a | 1.3 | 0.5 b | 0.3 a |
|  | **EE** | 2.45 | 5.0 | 27.2 a | 2.08 a | 20.1 | 2.5 b | 18.9 a | 8.8 | 6.7 | 13.8 b | 1.5 a | 1.0 | 0.7 a | 0.2 b |
|  | *p-value* | *0.441* | *0.226* | *<0.001* | *0.006* | *0.108* | *<0.001* | *0.003* | *0.655* | *0.882* | *<0.001* | *0.006* | *0.334* | *<0.001* | *<0.001* |
| **SR** | **161** | 2.91 a | 6.7 | 15.8 | 4.6 | 17.9 | 10.4 a | 13.3 | 7.1 | 9.2 a | 21.7 | 1.9 a | 1.6 a | 0.5 b | 0.3 a |
|  | **321** | 2.21 a | 5.0 | 21.8 | 5.0 | 15.5 | 4.2 b | 15.1 | 9.2 | 3.8 a | 25.4 | 1.5 a | 0.6 b | 0.6 a | 0.2 a |
|  | *p-value* | *0.01* | *0.215* | *0.168* | *0.831* | *0.565* | *0.005* | *0.461* | *0.414* | *0.034* | *0.313* | *0.02* | *0.002* | *0.001* | *0.01* |
| **P x SR** |  | *0.523* | *0.841* | *0.006* | *0.019* | *0.565* | *0.042* | *<0.001* | *0.141* | *0.244* | *0.865* | *0.378* | *0.647* | *0.167* | *0.161* |
|  |  |  |  |  |  |  |  |  |  |  |  |  |  |  |  |
| **USIrr20** |  |  |  |  |  |  |  |  |  |  |  |  |  |  |  |
| **P** | **ME** | 4.18 | 3.9 | 10.8 a | 13.3 a | 11.3 | 6.2 a | 13.8 | 7.5 | 9.2 | 27.9 a | 1.6 | 0.7 | 0.5 b | 0.3 a |
|  | **EE** | 4.33 | 3.0 | 23.7 a | 5.8 a | 15.4 | 0.0 a | 20.8 | 7.5 | 9.2 | 17.5 a | 1.5 | 0.6 | 0.7 a | 0.2 b |
|  | *p-value* | *0.441* | *0.187* | *0.001* | *0.013* | *0.235* | *<0.001* | *0.258* | *1.000* | *1.000* | *0.025* | *0.743* | *0.363* | *<0.001* | *<0.001* |
| **SR** | **161** | 4.51 | 4.4 | 20.8 | 7.5 | 15.0 | 2.5 | 18.3 | 5.8 ab | 10.0 | 20.0 | 1.5 | 0.5 | 0.6 | 0.2 |
|  | **215** | 4.39 | 3.7 | 18.3 | 10.0 | 7.5 | 5.0 | 20.8 | 8.3 ab | 9.2 | 20.8 | 1.6 | 0.6 | 0.6 | 0.2 |
|  | **269** | 3.96 | 2.9 | 13.3 | 9.2 | 14.2 | 0.8 | 12.5 | 12.5 a | 11.7 | 25.8 | 1.6 | 0.6 | 0.6 | 0.2 |
|  | **321** | 4.16 | 2.8 | 16.7 | 11.7 | 16.7 | 4.2 | 17.5 | 3.3 b | 5.8 | 24.2 | 1.6 | 0.7 | 0.6 | 0.3 |
|  | *p-value* | *0.178* | *0.255* | *0.496* | *0.806* | *0.268* | *0.356* | *0.58* | *0.036* | *0.313* | *0.79* | *0.971* | *0.865* | *0.458* | *0.063* |
| **P x SR** |  | *0.424* | *0.455* | *0.306* | *0.534* | *0.43* | *0.356* | *0.838* | *0.731* | *0.037* | *0.896* | *0.616* | *0.484* | *0.286* | *0.031* |
|  |  |  |  |  |  |  |  |  |  |  |  |  |  |  |  |
| **USDry20** |  |  |  |  |  |  |  |  |  |  |  |  |  |  |  |
| **P** | **ME** | 2.90 | 2.8 | 32.9 a | 5.0 a | 16.3 | 0.4 a | 16.7 | 12.1 | 5.8 | 10.8 | 1.3 a | 0.5 | 0.7 a | 0.19 a |
|  | **EE** | 3.09 | 2.5 | 15.4 a | 17.5 b | 14.2 | 5.4 a | 17.9 | 8.8 | 9.6 | 11.3 | 1.2 a | 0.4 | 0.5 b | 0.25 a |
|  | *p-value* | *0.575* | *0.611* | *0.002* | *<0.001* | *0.574* | *0.008* | *0.724* | *0.300* | *0.083* | *0.905* | *0.030* | *0.535* | *<0.001* | *0.001* |
| **SR** | **161** | 2.79 | 4.1 a | 31.7 a | 10.0 | 10.0 | 3.3 | 15.8 | 6.7 | 8.3 | 14.2 | 1.3 | 0.5 | 0.6 | 0.2 |
|  | **215** | 3.17 | 2.7 ab | 29.2 ab | 11.7 | 14.2 | 0.8 | 14.2 | 16.7 | 6.7 | 6.7 | 1.2 | 0.4 | 0.7 | 0.2 |
|  | **269** | 2.99 | 1.9 b | 18.3 b | 13.3 | 19.2 | 3.3 | 15.8 | 12.5 | 8.3 | 9.2 | 1.2 | 0.4 | 0.6 | 0.2 |
|  | **321** | 3.03 | 1.9 b | 17.5 b | 10.0 | 17.5 | 4.2 | 23.3 | 5.8 | 7.5 | 14.2 | 1.3 | 0.5 | 0.6 | 0.2 |
|  | *p-value* | *0.707* | *0.009* | *0.024* | *0.749* | *0.310* | *0.623* | *0.257* | *0.056* | *0.939* | *0.134* | *0.440* | *0.819* | *0.266* | *0.227* |
| **P x SR** |  | *0.85* | *0.820* | *0.122* | *0.276* | *0.519* | *0.581* | *0.611* | *0.003* | *0.013* | *0.363* | *0.493* | *0.743* | *0.749* | *0.381* |

^1^Planter type: Max Emerge (ME) and Exact Emerge (EE)

^2^Letter and ANOVA threshold. Different letters mean significant differences among treatments.

**Supplementary Table 2.** ANOVA table of ATEI sd (achieved versus targeted evenness index standard deviation) and seeding rate (SR) level effects on soybean yield (Mg ha^-1^). Low, medium, and high SR levels based on SR lower than 200 seeds m^-2^, between 200 and 300 seeds m^-2^, and greater than 300 seeds m^-2^, respectively.

| **SR level** | **Intercept**  **(SR level)** | **Slope**  **(ATEI sd)** |
| --- | --- | --- |
| **Low** | 3.91 | -1.26 |
| **Medium** | 3.86 | -0.732 |
| **High** | 4.01 | -1.022 |
| **Pr(>F)** | ATEI sd | <0.01 |
|  | SR level | 0.83 |
|  | ATEI sd xSR level | 0.26 |


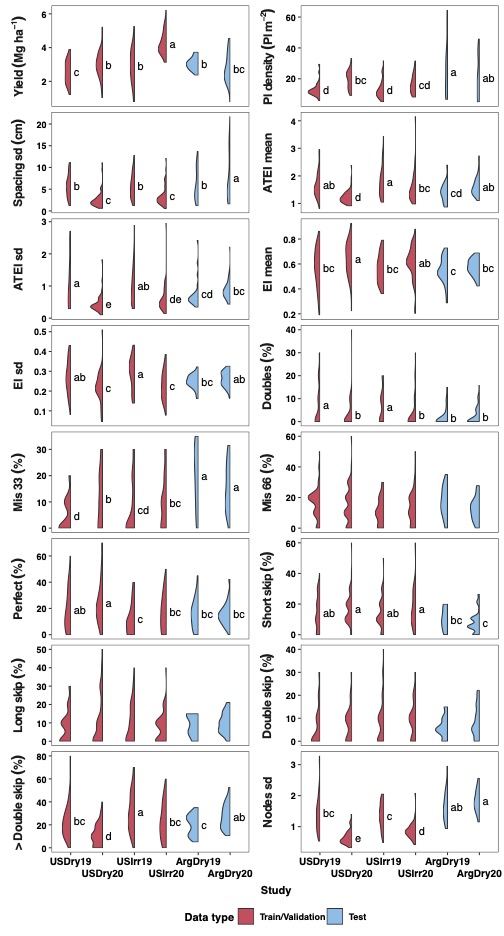


**Supplementary Figure 1.** Effect of site-year on yield, plant density, and all community-scale metrics of spatial uniformity (ATEI mean, ATEI sd, EI mean, EI sd, Doubles, Mis 33, Mis 66, Perfect, Short-skip, Long-skip, Double-skip and >Double-skip). Different colors denote site-year used as training (red) and testing (blue) data sets. Fisher’s LSD pair-wise comparisons was performed on significative variables. Means followed by the same letter are not significantly different at P = 0.05. References: ATEI sd = achieved versus targeted evenness index standard deviation, EI sd = evenness index standard deviation, Double skip = percentage of double skips plants, Mis 66 = percentage of plants misplaced by 66%, U.S.: United States, Arg: Argentina, Dry: dryland, Irr: Irrigated, 19 and 20: 2019 and 2020 calendar years.


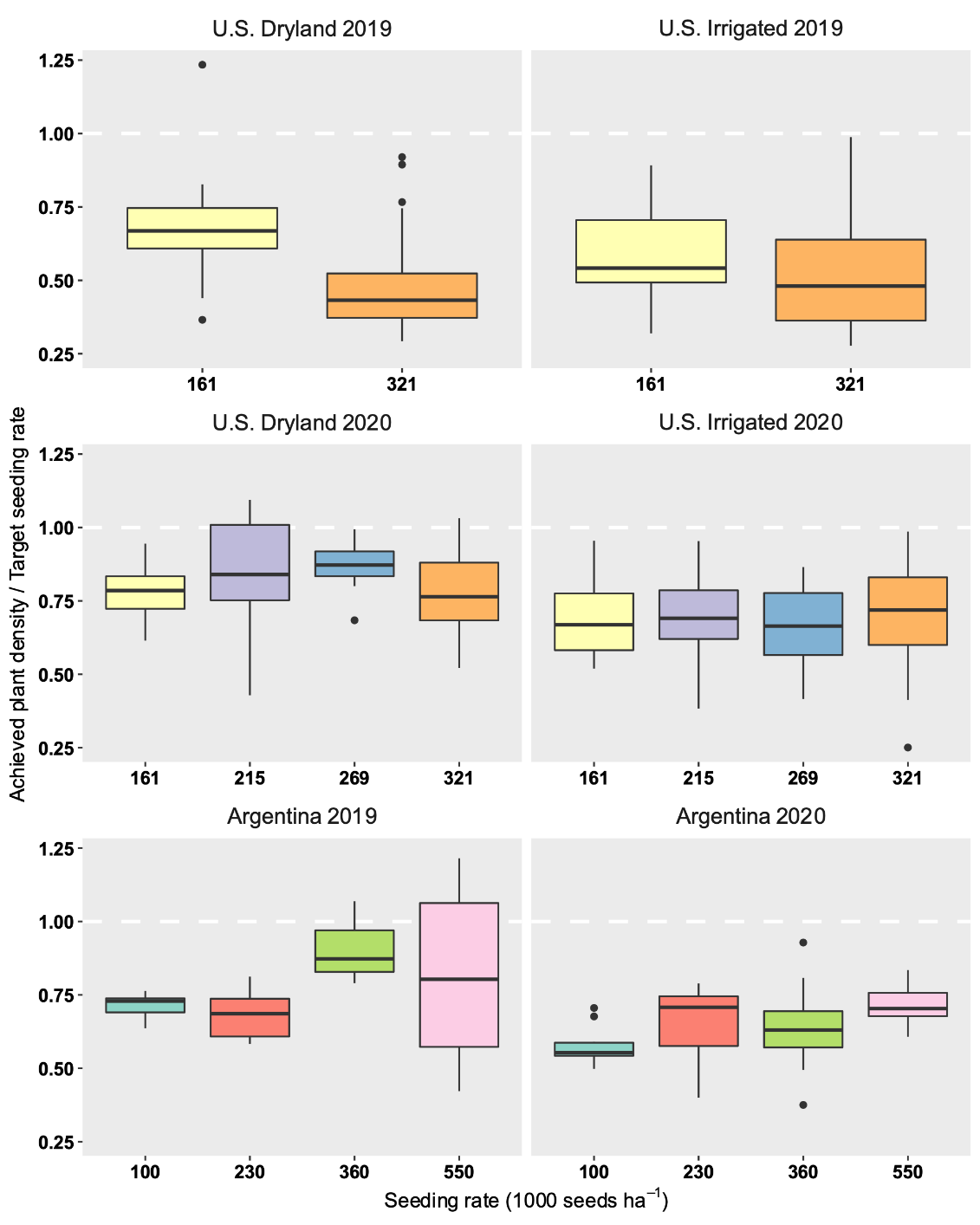


**Supplementary Figure 2.** Boxplots of relative density (achieved plant density to target seeding rate ratio) by seeding rate (1000 seeds ha^-1^), at each of the six studies analyzed.


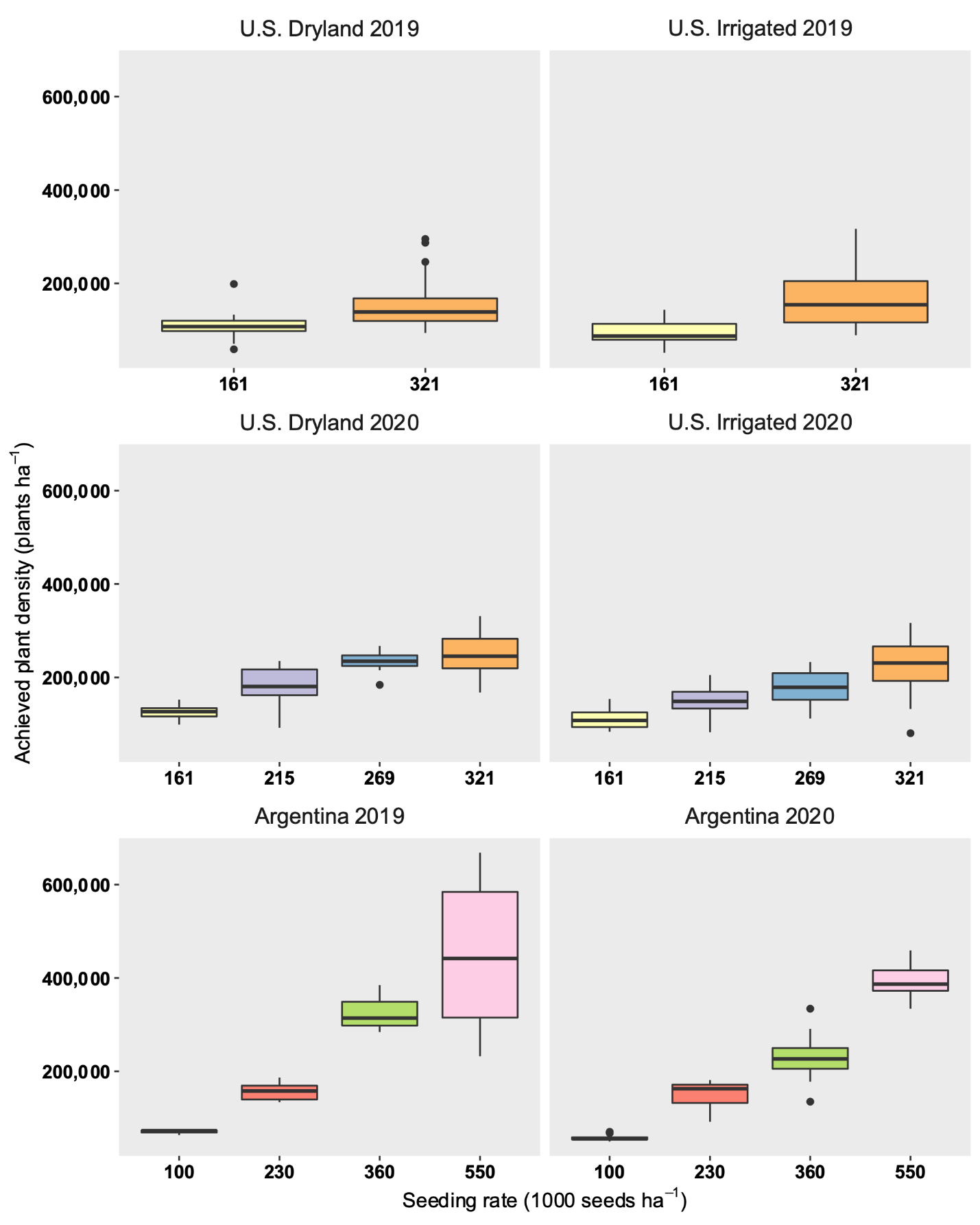


**Supplementary Figure 3**. Boxplots of Achieved plant density (plants ha^-1^) by seeding rate (1000 seeds ha^-1^), at each of the six studies analyzed.
